# Chemoradiotherapy followed by durvalumab in patients with unresectable advanced non‐small cell lung cancer: Management of adverse events

**DOI:** 10.1111/1759-7714.13394

**Published:** 2020-03-11

**Authors:** Yu Miura, Atsuto Mouri, Kyoichi Kaira, Ou Yamaguchi, Ayako Shiono, Kosuke Hashimoto, Fuyumi Nishihara, Shun Shinomiya, Tomoe Akagami, Yoshitake Murayama, Takanori Abe, Shin‐ei Noda, Shingo Kato, Kunihiko Kobayashi, Hiroshi Kagamu

**Affiliations:** ^1^ Department of Respiratory Medicine Comprehensive Cancer Center, International Medical Center, Saitama Medical University Saitama Japan; ^2^ Department of Radiation Oncology Comprehensive Cancer Center, International Medical Center, Saitama Medical University Saitama Japan

**Keywords:** Chemoradiotherapy, durvalumab, non‐small cell lung cancer, radiation pneumonitis, real‐world experience

## Abstract

**Background:**

Chemoradiotherapy followed by durvalumab is the standard treatment for the patients with local advanced non‐small cell lung cancer (NSCLC). There is a real‐world data about the management of adverse events, such as pneumonitis, according to the different institutions. Here, we present the experience regarding the management of adverse events after the initiation of durvalumab as daily practice.

**Methods:**

From July 2018 to August 2019, 41 patients with locally advanced NSCLC, who underwent chemoradiotherapy followed by durvalumab, were retrospectively analyzed in the study using our medical records.

**Results:**

The median age of patients was 72 years (range: 51–80 years). A total of 33 patients were male and eight were female, and 40 patients (98%) received a total radiation dose of 60 Gy as concomitant chemoradiotherapy. The median V20 for the entire cohort was 18.9% (range: 3.5–29.9). Any adverse events during chemoradiotherapy and durvalumab were observed in 32 patients (78.0%), while three patients (7.3%) experienced grade 3 toxicities. In total, 25 (61.0%) patients experienced pneumonitis, four (9.8%) thyroid dysfunction, three (7.3%) myopathy, two (4.9%) rash or eruption, one (2.4%) bowel disease and one (2.4%) malaise. Grade 3 pneumonitis, thyroid dysfunction and myopathy were observed in one (2.4%), one (2.4%) and one (2.4%), respectively. A total of 22 (53.7%) patients were unable to continue durvalumab due to pneumonitis. However, durvalumab was finally readministered to six patients.

**Conclusions:**

The adherence to lung dose constraints such as V20 as well as close treatment monitoring are a prerequisite for the management of pneumonitis during maintenance therapy with durvalumab.

## Introduction

Chemoradiotherapy is the standard treatment for the patients with locally advanced non‐small cell lung cancer (NSCLC). Durvalumab is a human IgG1 monoclonal antibody that blocks programmed death ligand 1 (PD‐L1) binding to programmed death 1 (PD‐1) and CD80.^1^ Clinical trials have revealed an antitumor activity in patients with several advanced solid tumors such as NSCLC.^2^ Recently, durvalumab has been widely administered as a maintenance therapy after platinum‐based concurrent chemoradiotherapy, based on the evidence of PACIFIC study.^3^ Antonia *et al*. reported that durvalumab as consolidation after chemoradiotherapy yielded a significantly longer overall survival than placebos,^4^ suggesting the potential of long‐term survival. However, interstitial lung injury (ILD) has been known to occur as an immune‐related adverse event (irAE) after the administration of anti‐PD‐1/PD‐L1 antibodies; moreover, radiation pneumonitis after chemoradiotherapy frequently occurs in patients with NSCLC. Thus, we have experienced the increased frequency of pneumonitis or radiation pneumonitis after chemoradiotherapy followed by durvalumab, as compared with chemoradiotherapy alone. The results of the PACIFIC trial demonstrated that pneumonitis was observed in 33.9% and 24.8% of patients, with and without durvalumab, after chemoradiotherapy, respectively,^3^ suggesting an increase in frequency of pneumonitis as a result of the additional administration of durvalumab. In daily practice, the management of pneumonitis is slightly different according to the patient's situation and physician's discretion. Therefore, we need to elucidate the real‐world data regarding the efficacy, adverse events and management of pneumonitis after chemoradiotherapy followed by durvalumab.

Recently, a retrospective analysis reported that 19 (23%) of 82 patients with stage III NSCLC, who were eligible at the initiation of chemoradiotherapy, became ineligible after chemoradiotherapy according to the registered criteria of PACIFIC trial, and old age, male gender and radiation therapy with the volume of the lung that received more than 20 Gy (V20), more than 35% were closely related to the ineligibility after chemotherapy.^5^ In their study, ineligible patients for the PACIFIC study had a trend toward shorter survival than eligible patients.^5^ Sakaguchi *et al*. also described a retrospective study to assess the eligibility of patients with unresectable stage III NSCLC who were able to receive durvalumab after chemoradiotherapy based on the PACIFIC trial criteria.^6^ In their study, radiation pneumonitis of any grade and grade 2 or more occurred in 54 (73.9%) and 12 (16.4%) of 73 patients after chemoradiotherapy, respectively. Including the other adverse events, 22 patients (30.1%) were identified as ineligible to receive durvalumab according to the criteria of the PACIFIC study. However, little is known about the tolerability and feasibility of durvalumab in patients who were treated with chemoradiotherapy outside clinical trials. Here, we present our experience of the management, feasibility and tolerability of durvalumab as consolidation therapy after chemoradiotherapy in patients with unresectable locally advanced NSCLC.

## Methods

### Patients

We retrospectively examined our medical records at Saitama Medical University, International Medical Center, and selected the patients with unresectable locally advanced NSCLC who received durvalumab as consolidation therapy after concurrent chemoradiotherapy in clinical practice. From July 2018 to August 2019, 53 patients were treated with chemoradiotherapy for unresectable locally advanced NSCLC. Of these 53 patients, 12 were unable to be treated with durvalumab because of progressive disease, pneumonitis and reduced general condition and 41 received durvalumab as consolidation therapy after chemoradiotherapy. Thus, a total of 41 patients were eligible for further analysis in our retrospective study. This study was approved by the institutional ethics committee of the Saitama Medical University International Medical Center.

### Systemic treatment

Durvalumab was intravenously administered at 10 mg/kg every two weeks. The chemotherapeutic regimens of chemoradiotherapy were different according to the physician's discretion. Out of 41 patients, 14 were treated with daily carboplatin (CBDCA) alone, 18 weekly CBDCA plus paclitaxel, four with cisplatin (CDDP) plus docetaxel (days 1 and 8) and five with the others. Complete blood cell count, differential count, routine chemistry measurements, physical examination, and toxicity assessment were performed on a weekly basis. Acute toxicity was graded using the Common Terminology Criteria for Adverse Events version 4.0. Tumor response was evaluated according to the Response Evaluation Criteria in Solid Tumors (RECIST) version 1.1.^7^

### Radiotherapy setting

Radiation as concurrent phase with induction chemotherapy was administered using 10 MV X‐rays in 2 Gy per fraction. The prescribed total dose was 60 Gy in 30 fractions. Computed tomography (CT) scans with 2.5 mm thickness were used for the treatment planning. All treatment plans were created using a commercially available treatment planning system (Xio version 6.2, Elekta, Inc., Stockholm, Sweden) and the dose calculation algorithm was convolution/superposition. CT images for treatment planning were obtained under normal breathing conditions, and a four‐dimensional CT was also obtained to identify tumor respiratory motion. The gross tumor volume (GTV) was contoured according to the primary tumor and nodal involvement determined by CT, and 2‐[^18^F]‐fluoro‐2‐deoxy‐D‐glucose (^18^F‐FDG) positron emission tomography (PET)/CT information. GTV on both inspiratory phase and expiratory phase CT image were contoured and defined as internal target volume (ITV). The clinical target volume (CTV) consisted of a volume with 5–10 mm margin from ITV and prophylactic lymph node regions included the ipsilateral hilum and the mediastinum. The dose constraints to the organs at risk included the following: V20 total lung 35% and maximum dose to the spinal cord 50 Gy; volume of the heart that received more than 60 Gy was less than 33%, volume of the heart that received more than 45 Gy was less than 67%, and volume of the heart that received more than 40 Gy was less than 100%; mean dose to the esophagus was less than 34 Gy.

### Statistical analysis

Statistical significance was indicated by *P* < 0.05. Fisher's exact tests were used to examine the association between two categorical variables. The Kaplan‐Meier method was used to estimate survival as a function of time, and survival differences were analyzed by log‐rank tests. Progression‐free survival (PFS) was defined as the time from the initiation of durvalumab therapy to tumor recurrence or death from any cause, while overall survival (OS) was defined as the time from the initiation of durvalumab therapy to death from any cause. Statistical analyses were performed using GraphPad Prism 4 (Graph Pad Software, San Diego, CA, USA) and JMP 8.0 (SAS Institute Inc., Cary, NC, USA).

## Results

### Patient demographics and durvalumab administration status

**Figure 1:**
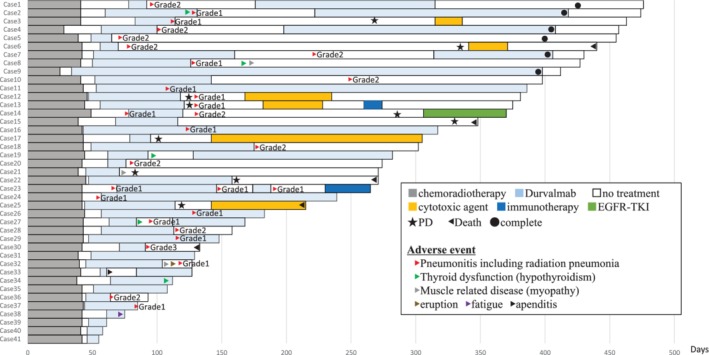
Treatment duration of chemoradiotherapy and durvalumab in all patients.

Patient demographics are listed in Table 1. The median age was 72 years (range: 51–80 years). A total of 33 were male and eight were female, and 24 patients (80%) had a performance status (PS) of 0 and 17 were one in PS. The histological types of adenocarcinoma (AC), squamous cell carcinoma (SCC) and not otherwise specified (NOS) were 21 (51%), 15 (37%) and five (12%) of all patients, respectively. The PD‐L1 expression was less than 1% in 12 patients (29%), 1% to 49% in 11 patients (27%) and more than 50% in nine patients (22%), respectively. A total of 40 patients (98%) received a radiation dose of 60 Gy. Figure 1 shows the clinical course of chemoradiotherapy followed by durvalumab in all patients. A total of 22 (53.7%) patients were unable to continue to receive durvalumab due to pneumonitis after the initiation of durvalumab. A total of 16 (39.0%) of the patients could not continue durvalumab treatment because of immune‐mediated pneumonitis. In six patients, durvalumab was readministered after a break. A total of 10 (24.3%) patients had experienced progressive disease (PD) within one year from the start of durvalumab. The median interruption of durvalumab was 94.5 days.

**Table 1:** Demographics of patients

Different variables	*N* = 41 (%)
Age	
Median years (range)	72 years (51 to 80 years)
Gender	
Male/Female	33 (80)/8 (20)
ECOG performance status	
0/1	24 (58)/17 (42)
Smoking history	
Yes/No	33 (80)/8 (20)
Histological type	
Adeno/SQ/NOS	21 (51)/15 (37)/5 (12)
Clinical disease stage	
IIIA/IIIB/IIIC/others	18 (44)/15 (37)/2 (5)/6 (14)
Mutation status	
EGFR/ALK/ROS1/none	5 (12)/0 (0)/1 (3)/35 (85)
TPS by PD‐L1	
1% </1–49%/50%≥/unknown	12 (29)/11 (27)/9 (22)/9 (22)
Total radiation dose	
60 Gy/30 Fr	40 (98)
54 Gy/25 Fr	1 (2)
Chemotherapeutic regimen	
CBDCA+PTX	18 (44)
CBDCA	14 (34)
CDDP+DTX	4 (10)
CDDP+TS‐1	3 (8)
CBDCA+DTX	1 (2)
CDDP+ETP	1 (2)
V20 (%)	
Median value (range)	
Interval from the end of irradiation to the start of Durvalumab	
Median days (range)	11 (1 to 42)
≤14 days/>14 days	25 (61)/16 (39)

Adeno, adenocarcinoma; ALK, anaplastic lymphoma kinase; CBDCA, carboplatin; CDDP, cisplatin; DTX, docetaxel; ECOG, Eastern Cooperative Oncology Group; EGFR, epidermal growth factor receptor; ETP, etoposide; NOS, not otherwise specified; PD‐L1, programmed‐death ligand‐1; PTX, paclitaxel; ROS1, proto‐oncogene tyrosine‐protein kinase; SG, squamous cell carcinoma; TPS, tumor proportional score.

Six patients (14.6%) completed durvalumab for one year and 17 are still on maintenance therapy. In 18/41 (43.9%), durvalumab was withdrawn due to progressive disease (*n* = 10; 55.9%), adverse events (*n* = 7; 38.9%) and other (*n* = 1; 5.5%). Dose constraints for lung, spinal cord, heart and esophagus were met in all patients.

### Toxicity profiles during administration of durvalumab

Table 2 shows adverse events after the initiation of durvalumab, compared with the Japanese cohorts in the PACIFIC trial. Any adverse events were observed in 36 patients (97.8%), and three patients (7.3%) experienced grade 3 toxicities. Of all patients, 25 (61.0%) experienced pneumonitis, four (9.8%) thyroid dysfunction, three (7.3%) myopathy, two (4.9%) rash or eruption, one (2.4%) bowel disease and one (2.4%) malaise. Grade 3 pneumonitis, thyroid dysfunction and myopathy were observed in one (2.4%), one (2.4%) and one (2.4%), respectively.

**Table 2:** Adverse events after initiation of durvalumab

	The present study *N* = 41		PACIFIC study Japanese cohort *N* = 72	
Different factors	All grade (%)	Over grade 3 (%)	All grade (%)	Over grade 3 (%)
Pneumonitis				
All grade	25 (61.0%)	1 (2.4%)	53 (73.6%)	5 (6.9%)
Grade 1	13 (31.7%)		31 (43.1%)	
Grade 2	11 (26.8%)		17 (23.6%)	
Grade 3	1 (2.4%)		4 (5.6%)	
Grade 4	0 (0%)		0 (0%)	
Thyroid dysfunction	4 (9.8%)	1 (%)	11.1%	0%
Myopathy	3 (7.3%)	1 (%)	6.9%	0%
Rash or eruption	2 (4.9%)	0 (0%)	13.9%	0%
Bowel disease	1 (2.4%)	0 (0%)	1.1%	0%
Malaise	1 (2.4%)	0 (0%)	8.3%	0%

### Clinical profiles of pneumonitis related to radiation or durvalumab

**Figure 2:**
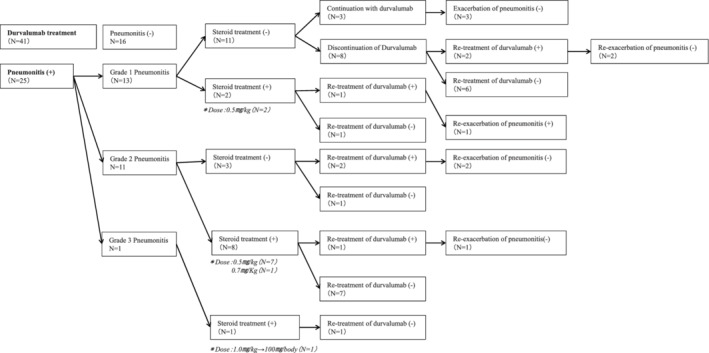
Clinical course of 25 patients with pneumonitis after administration of durvalumab.

Table 3 shows the clinical features of 25 patients (61.0%) who experienced pneumonitis. The patients who experienced grade 1, 2 and 3 pneumonitis were 13 (52.0%, 13/25), 11 (26.8%, 11/25) and one (4.0%, 1/25), respectively. The median value of V20 (%) in all patients (*n* = 41) was 18.9%, ranging from 3.5% to 29.9%. The median V20 was 20.1% (range: 10.4%–29.9%) in the 25 patients with pneumonitis, while in the other 16 was tendentially lower (*P* = 0.35) with a median value of 17.7% (range: 3.4%–29.2%). We analyzed the difference in clinical features between the patients with grade 1 and grade 2 pneumonitis. No statistically significant difference in the median interval from the initiation of durvalumab to the occurrence of pneumonitis was observed between the patients with grade 1 and grade 2 pneumonitis (*P* = 0.05; 69 days vs. 78 days). Figure 2 shows the clinical and therapeutic management according to the grading of pneumonitis. Of 13 patients with grade 1 pneumonitis, 11 (84.6%) needed no steroid therapy, durvalumab was discontinued in eight (61.5%), and one of three (23.1%) who received retreatment with durvalumab displayed re‐exacerbation of pneumonitis. There were two patients who received corticosteroid therapy with a dose commencing at 0.5 mg/kg because of potentially having immune‐related pneumonitis, and one patient who was retreated with durvalumab experienced re‐exacerbation of pneumonitis. Of 11 patients with grade 2 pneumonitis, eight received corticosteroid therapy with a starting dose of 0.5–0.7 mg/kg, and durvalumab was discontinued in seven, and two of the three patients who needed no corticosteroid therapy received durvalumab again after the cessation of treatment, without exacerbation of pneumonitis. Grade 1 or 2 pneumonitis improved in all patients who received corticosteroid therapy of 0.5 mg/kg.

**Table 3:** Clinical features of 25 patients who experienced pneumonitis

Case	Pneumonitis before durva.	V20 (%)	Interval from durva.to pneumonitis	Grade	Dyspnea	Desaturation	Fever	Shadow outside RT therapy	CRP	Continuation of Durvalumab	Steroid therapy	Pneumonitis after steroid	Retreatment with Durvalumab
Case 1	—	23.99	58	2	—	—	—	—	0.6	—	N/A	—	Yes
Case 2	—	14.56	76	1	—	—	—	Yes	0	—	N/A	—	Yes
Case 3	—	23.07	38	1	—	—	—	—	1.7	—	0.5 mg/kg	Improve	—
Case 4	—	27.89	72	2	—	—	—	Yes	3.3	—	N/A	—	Yes
Case 5	—	24.28	26	2	Yes	—	—	Yes	2.1	—	0.5 mg/kg	Improve	—
Case 6	—	—	43	2	Yes	Yes	—	Yes	3.5	—	0.5 mg/kg	Improve	—
Case 7	—	16.13	143	2	Yes	Yes	Yes	Yes	11.1	—	0.5 mg/kg	Improve	Yes
Case 8	—	14.19	85	1	—	—	—	—	6.2	—	N/A	—	—
Case 10	—	18.89	175	2	—	—	—	—	5.5	—	0.5 mg/kg	Improve	—
Case 11	—	29.18	53	1	—	—	—	—	0.5	Yes	N/A	—	—
Case 12	—	18.66	84	1	—	—	—	—	0.4	—	N/A	—	—
Case 13	—	12.36	77	1	—	—	—	—	16	—	N/A	—	—
Case 14	Grade 1	22.45	71	2	—	—	—	—	N/A	—	N/A	—	—
Case 16	—	20.67	86	1	—	—	—	—	0	Yes	N/A	—	—
Case 18	—	19.62	132	2	Yes	Yes	Yes	Yes	11.6	—	0.5 mg/kg	Improve	—
Case 20	—	25.66	48	2	Yes	Yes	—	—	5	—	0.5 mg/kg	Improve	—
Case 23	Grade 1	24.03	69	1	—	—	—	—	8.9	—	0.5 mg/kg	Improve	Yes
Case 26	—	19.52	93	1	—	—	—	—	0.9	Yes	N/A	—	—
Case 27	—	15.09	56	1	—	—	—	—	0.1	—	N/A	—	Yes
Case 28	—	29.93	68	2	Yes	Yes	—	Yes	0.7	—	0.7 m/kg	Improve	—
Case 29	—	10.46	67	1	—	—	—	—	1.5	—	N/A	—	—
Case 30	CPFE	17.76	49	3	Yes	Yes	—	Yes	17	—	1.0 mg/kg	Improve	—
Case 32	—	17.46	83	1	—	—	—	—	1.4	—	N/A	—	—
Case 36	—	29.18	30	2	Yes	—	—	—	0.3	—	0.5 mg/kg	Improve	—
Case 37	—	20.66	42	1	—	—	—	—	0.4	—	N/A	—	—

CPFE, chronic pulmonary fibrotic emphysema; durva., durvalumab; N/A, not applicable; RT, radiation.

Two (33.3%) of six patients who received retreatment with durvalumab experienced re‐exacerbation of pneumonitis.

### Survival and locoregional control

**Figure 3:**
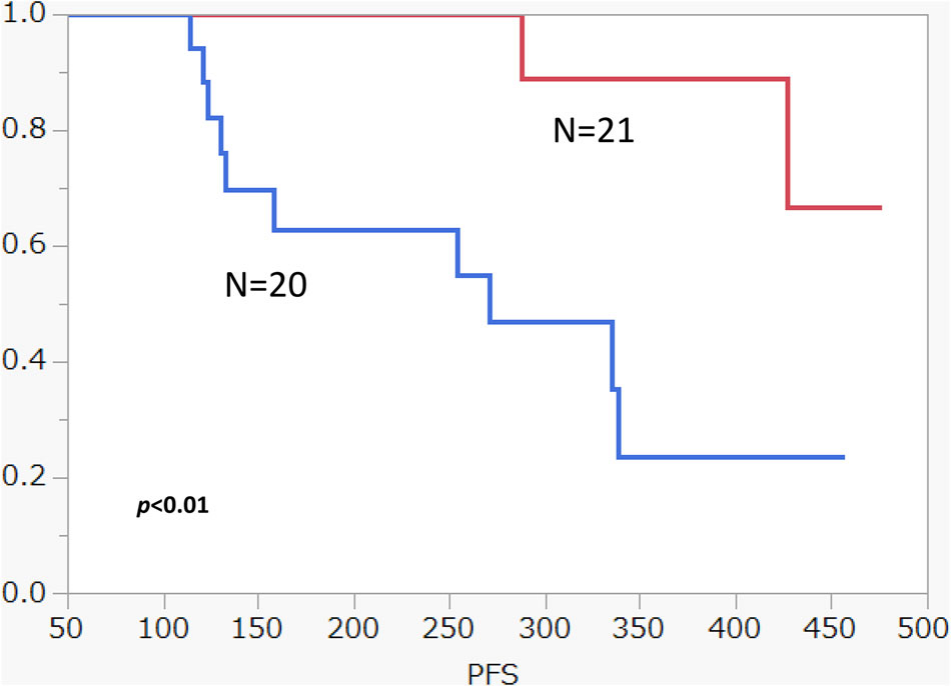
PFS in patients with adenocarcinoma compared to those with other histologies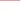 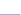.

Of all the patients in the study, 12 experienced a recurrence and seven died because of progressive disease. The median PFS and OS from the initiation of chemoradiotherapy were 423 days and 463 days, respectively. The median follow‐up period was 271 days. In order to calculate PFS, the patients were stratified by the occurrence of pneumonitis. By univariate analysis, no statistically significant difference in the PFS was observed between the patients with and without pneumonitis (*P* = 0.24) and between those with grade 1 and 2 pneumonitis (*P* = 0.18), respectively. Although the univariate analysis in the PFS according to gender, PS and V20 (%) showed no significant difference, the patients with adenocarcinoma displayed a significantly better PFS than those with nonadenocarcinoma (*P* < 0.01) (Fig 3). We then compared clinically different variables between the patients with AC and non‐AC. (Table S1, online only). The frequency of PS of 0 was significantly higher in the patients with AC than in those with non‐AC, but not that of gender, pneumonitis and V20 (%). Also, there was no statistically significant difference in the PFS between the patients where it was more, or less than, 14 days from the end of thoracic radiation to the initiation of durvalumab (*P* = 0.77).

## Discussion

To our knowledge, this is the first study to present real‐world data from a single institution of the administration of chemoradiotherapy followed by durvalumab in patients with unresectable advanced NSCLC. In our study, the frequency of pneumonitis after administration of durvalumab was 61% of all patients, which was in the same range as the 60% in the Japanese cohort of the PACIFIC study. Although there was no significant difference in the value of V20 (%) between the patients with and without pneumonitis, that of V20 (%) in the patients with grade 2 pneumonitis was significantly higher than that with grade 1 pneumonitis. All patients who received corticosteroids of 0.5 mg/kg experienced an improvement in their pneumonitis. According to the practical guidelines regarding the management of irAEs, it is recommended that oral systemic steroids with a dosage of 0.5 to 1.0 mg/kg/day should be administered to patients who experience grade 1 or 2 toxicities.^8^ Although it remains unclear whether corticosteroids of 1.0 mg/kg are better for the improvement of grade 1 or 2 toxicities than that of 0.5 mg/kg, our experience suggests that a dosage of 0.5 mg/kg/day is appropriate as a starting dose of corticosteroid for patients with a grade 1 or 2 pneumonitis.

In the PACIFIC trial, the frequency of any pneumonitis grade in patients with and without durvalumab was 33.9% and 24.8%, respectively. Pneumonitis grade 3 or higher occurred in 3.4% and 2.6%, respectively. Moreover, immune‐mediated adverse events of any grade were seen in 24.2%, while higher grades (ie, grades 3 and 4) occurred in 3.4% of the patients with durvalumab treatment.^3^, ^8^ The data in this study suggested that durvalumab exhibited manageable toxicities after chemoradiotherapy. Although the results of the PACIFIC study demonstrated that glucocorticoids were administered to approximately 15% of patients with adverse events during the administration of durvalumab, it remains unclear how glucocorticoids were administered according to the grading of pneumonitis, regarding the therapeutic dosage of glucocorticoids, the correlation between V20 and the occurrence of pneumonitis, discontinuation of durvalumab and retreatment. In our study, approximately 60% of the patients receiving durvalumab had experienced any grade pneumonitis; however, the therapeutic dosage of glucocorticoids 0.5 mg/kg was actually sufficient to control the condition. If grade 2 pneumonitis occurs, it may be better to consider the cessation of durvalumab, although it is sometimes difficult to differentiate radiation‐pneumonitis from drug‐induced pneumonia. Little is known about the therapeutic significance of retreatment with durvalumab after its discontinuation. We are of the opinion that retreatment with durvalumab should be reconsidered according to individual conditions. Further studies are warranted to elucidate the optimal management of pneumonitis during the administration of durvalumab using larger sample sizes.

The current study has several limitations. First, the sample size is small, which is a major bias. Nevertheless, our data may gain significance in light of the fact that until now hardly any real‐world data on the toxicity profile of durvalumab outside clinical trials have been published. Second, the follow‐up period is immature, and therefore our survival analysis was an exploratory investigation. In the present study, the patients with AC achieved a significantly better PFS than those with other histologies, which is a result that may have been biased by PS. Further evaluation is warranted to present the survival data with a mature follow‐up period.

In conclusion, the results regarding the adverse events in our study were similar to those of the PACIFIC study. This means that the current study provides real‐world evidence that durvalumab can be safely administered in daily clinical practice. As more than half of patients experienced pneumonitis during the administration of durvalumab, it is critical to make a careful decision whether durvalumab should be discontinued. The adherence to lung dose constraints such as V20, as well as close treatment monitoring, are a prerequisite for the management of pneumonitis during maintenance therapy with durvalumab. Further large‐scale studies are necessary to investigate the impact of durvalumab maintenance therapy on survival.

## Disclosure

A.M., K.K., and H.K. received research grants and a speaker honorarium from Ono Pharmaceutical Company, Bristol‐Myers Company and AstraZeneca company. All remaining authors have declared no conflicts of interest.

## Supporting information


**Table S1.** Comparison of AC and non‐AC regarding different variables.
