# Supplementary material for: Chemoradiotherapy followed by durvalumab in patients with unresectable advanced non‐small cell lung cancer: Management of adverse events
Source: Thorac Cancer. 2020 Mar 11;11(5):1280–7. doi: 10.1111/1759-7714.13394 (PMC7180558; doi:10.1111/1759-7714.13394)
Supplement: Supplementary file 1 — Table S1. Comparison of AC and non‐AC regarding different variables. [file TCA-11-1280-s001.docx]

**Table A1. Comparison of AC and non-AC regarding different variables**

| **Variables** | **Total**  (n=41) | **AC**  (n=21) | **Non-AC**  (n=20) | *p*-value |
| --- | --- | --- | --- | --- |
| **Gender**  Male / Female | 33 / 8 | 15 / 6 | 18 / 2 | >0.99 |
| **PS**  O / 1 | 24 / 17 | 16 / 5 | 8 / 12 | **0.02** |
| **Pneumonitis**  Yes / No | 25 / 16 | 13 / 8 | 12 / 8 | >0.99 |
| **V20 (%)**  18.9 > / ≤ 18.9 | 20 / 21 | 11 / 10 | 9 / 11 | 0.75 |

Abbreviation: AC, adenocarcinoma; PS, performance status.
